# Impact of Shale Gas Development on Water Resources: A Case Study in Northern Poland

**DOI:** 10.1007/s00267-015-0454-8

**Published:** 2015-04-16

**Authors:** Ine Vandecasteele, Inés Marí Rivero, Serenella Sala, Claudia Baranzelli, Ricardo Barranco, Okke Batelaan, Carlo Lavalle

**Affiliations:** 1 Institute for Environment and Sustainability (IES), Joint Research Centre of the European Commission, Ispra, Italy; 2 Department of Hydrology and Hydraulic Engineering, Vrije Universiteit Brussel, Brussels, Belgium; 3 School of the Environment, Flinders University, Adelaide, Australia

**Keywords:** Land use modeling, Water consumption, Shale gas, Environmental impact

## Abstract

Shale gas is currently being explored in Europe as an alternative energy source to conventional oil and gas. There is, however, increasing concern about the potential environmental impacts of shale gas extraction by hydraulic fracturing (fracking). In this study, we focussed on the potential impacts on regional water resources within the Baltic Basin in Poland, both in terms of quantity and quality. The future development of the shale play was modeled for the time period 2015–2030 using the LUISA modeling framework. We formulated two scenarios which took into account the large range in technology and resource requirements, as well as two additional scenarios based on the current legislation and the potential restrictions which could be put in place. According to these scenarios, between 0.03 and 0.86 % of the total water withdrawals for all sectors could be attributed to shale gas exploitation within the study area. A screening-level assessment of the potential impact of the chemicals commonly used in fracking was carried out and showed that due to their wide range of physicochemical properties, these chemicals may pose additional pressure on freshwater ecosystems. The legislation put in place also influenced the resulting environmental impacts of shale gas extraction. Especially important are the protection of vulnerable ground and surface water resources and the promotion of more water-efficient technologies.

**Electronic supplementary material:**

The online version of this article (doi:10.1007/s00267-015-0454-8) contains supplementary material, which is available to authorized users.

## Introduction

There is increasing interest in the development of shale gas as a potential energy source in Europe. Resource estimates have been made for several member states (USDE 2011; Pearson et al. 2012), and exploration is on-going. Due to the low permeability of shale, alternative technologies are applied to increase the recovery rate of the gas. The resource is currently exploited by horizontal drilling of the shale formations to increase borehole contact and high-volume hydraulic fracturing (fracking) to stimulate migration of the gas through the shale. Fracking involves high pressure pumping of fluid through perforations in the well casing in order to produce hydrofractures which propagate through the surrounding shale (King 2012). There are several aspects related to the exploitation of shale gas which may be of concern. These include the occupation of large areas of land (Slonecker et al. 2012; Drohan et al. 2012a; Baranzelli et al. 2014), pollution (Bunch et al. 2014; Moore et al. 2014), impacts on biodiversity (Souther et al. 2014; Brittingham et al. 2014; Northrup and Wittemyer 2013; Kiviat 2013), and possibly seismic triggering (Rutqvist et al. 2013; Geny 2010). For a more detailed review of the available literature, see Kavalov and Pelletier (2012).

In this article, we focus on the possible impact of shale gas extraction by hydraulic fracturing on water resources (Vengosh et al. 2014; Mauter et al. 2014). The consumption of water involved in hydraulic fracturing may place additional pressure on freshwater resources (Arthur et al. 2010), as well as causing potential contamination thereof (Rahm and Riha 2012; Rahm et al. 2013). The competition for freshwater resources in densely populated areas remains an issue, even though some studies claim that energy production using shale gas can actually be more efficient in terms of water use than conventional natural gas (Scott et al. 2011; Mantell 2009). Besides the environmental concerns, the availability of freshwater resources may be a major restriction to companies wanting to extract shale gas commercially, especially where resources are already limited (Mangmeechai et al. 2013).

Four scenarios of shale gas extraction were modeled for our study site using the LUISA modeling platform. The main variables taken into account in the scenario definitions were the technology used, land and water requirements, and the legislation which may be put in place. Several scenarios were used to allow assessment of the range of possible impacts on the freshwater resources available.

Fracking fluid predominately consists of fresh water combined with sand and a variety of chemical additives including corrosion inhibitors, biocides, thickeners, and friction reducers (Arthur et al. 2009; Centner 2013). The impact on water quality will depend on various factors, including the chemical composition of the fracking water, the geology, and the technology used (Abbasi et al. 2014). To date, most studies of the potential environmental impacts of shale gas development have focused on assessing greenhouse gas emissions associated with shale gas production activities (Jiang et al. 2011, Howarth et al. 2011, Weber and Clavin 2012), drinking water quality effects (Osborn et al. 2011; Gross et al. 2013; USEPA 2012a), or regional air quality (McKenzie et al. 2012, Bunch et al. 2014). Entrekin et al. (2011) highlight that the data required to fully understand potential threats to surface water are currently lacking. Rozell and Reaven (2012) studied five pathways of water contamination, assessing the probability of occurrence of water pollution and also advocating the need for further detailed studies. Although there are several studies investigating the nature and magnitude of environmental and human health effects due to chemicals released as a result of shale gas development (Adams 2011; Adgate et al. 2014; Bamberger and Oswald 2012; Hill 2012; Wang et al. 2013; Drohan et al. 2012b), there is still no consensus on the subject. We therefore conducted a screening-level risk assessment of a wide variety of chemicals potentially used in fracking in order to better understand their physicochemical properties, potential fate in the environment, and the associated risk for freshwater.

In the following sections, we introduce the study area and explain the methodology used, including the scenarios adopted for the analysis, the indicators used to assess water demands, and the screening-level risk assessment. The results are then presented and discussed in light of management implications.

### Study Area

**Fig. 1:**
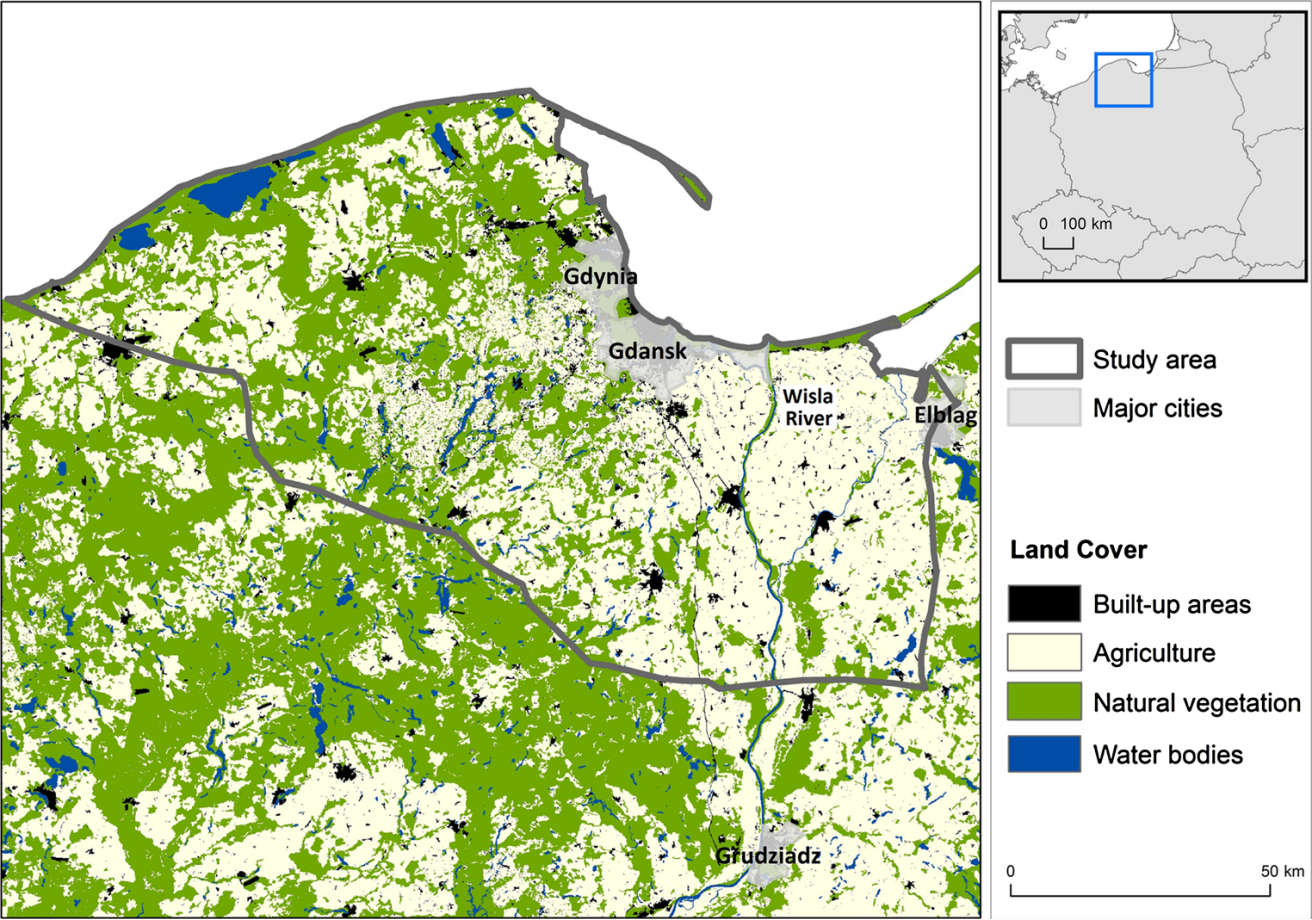
Map of the study area within Poland, indicating the land cover and shale gas exploration wells present

We looked at a specific case study in Northern Poland where the presence of notable shale gas resources has been confirmed (PGI 2012), and which was deemed the most suitable site for shale gas extraction in Poland in a previous study (Lavalle et al. 2013). The estimated total available shale gas resources within our study area are 386 Bcm (Baranzelli et al. 2014). At the time of writing, exploration drilling is permitted in Poland, but as yet no large-scale exploitation of the resource is being carried out. Figure 1 shows the land use, major cities, and location of several known shale gas exploration wells within and around the study site. The cities of Gdynia, Gdansk, and part of Elblag fall within the study area, as well as several important water bodies along the northern coast, and the Wisla River in the east. The main land uses are agriculture and forest, and there are several national parks situated within the study area.

**Fig. 2:**
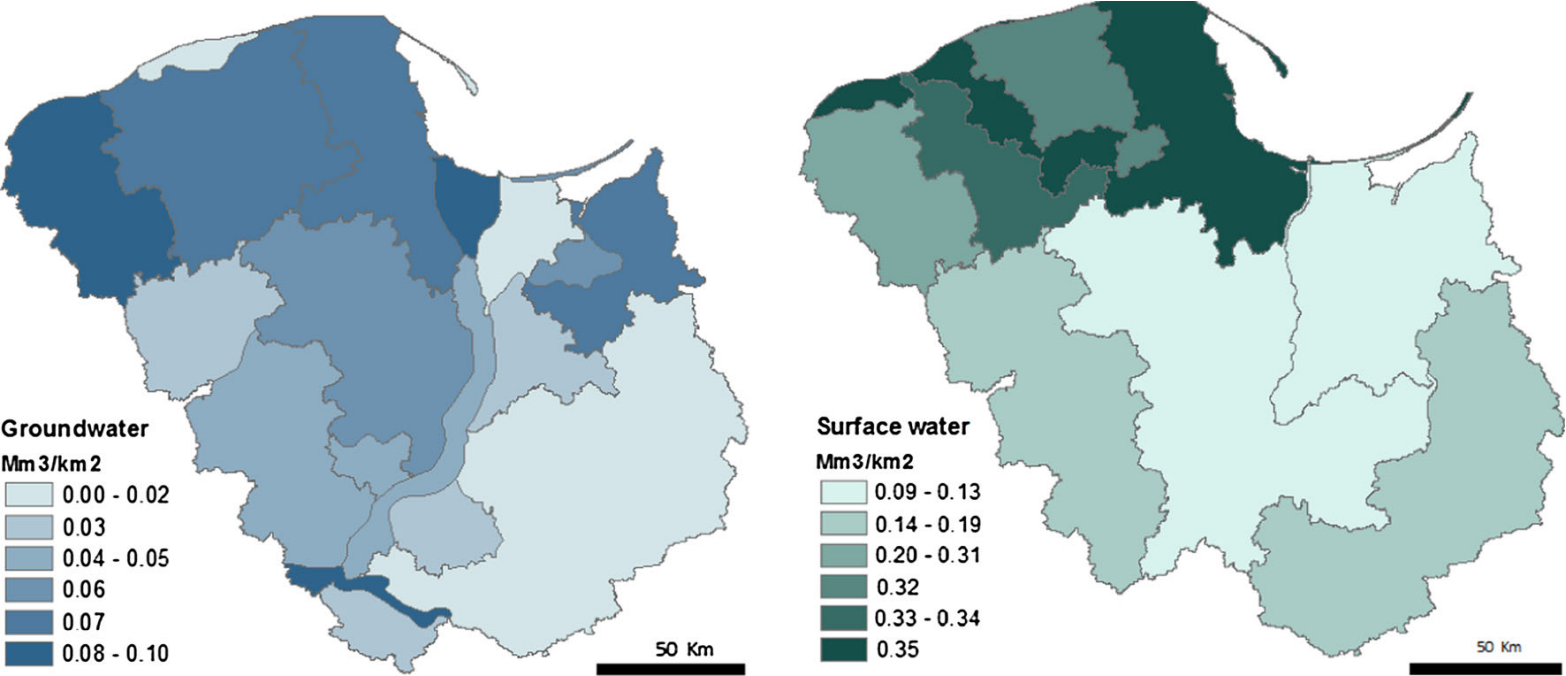
Freshwater available for use from groundwater and surface water resources in total millions of m^3^ per km^2^ for the year 2012 (Data Source: Polish Hydrogeological Survey)

The Polish Hydrogeological Survey provides detailed information on ground and surface water resources (PHS 2012). The groundwater resources available for development are given in thousands of m^3^ per day per hydrogeographical region. The average available surface water per sub-catchment was estimated based on the total flow within a catchment over a year. These data were used to represent the available water resources in our study area, as shown in Fig. 2.

According to these estimates, there is substantially more surface water available than groundwater. Groundwater availability is greatest in the northwest and in the regions surrounding the Wisla estuary. Surface resources are also greatest around the Wisla estuary, with much lower flows toward the centre and south of our study area.

## Methodology

### Shale Gas Extraction Scenarios

We assessed several scenarios of possible future shale gas development in the region for the period 2015–2030. These scenarios are defined in detail in Baranzelli et al. (2014), and include two technological scenarios (relatively higher and lower expected environmental impact) and two legislative scenarios (representing the current legislation in place, and a more restrictive framework). The scenarios were used firstly to determine the most suitable locations for shale gas exploration, and then to allocate the well pads in 5-year time steps using a land use model (EUCS100, Lavalle et al. 2011).

#### Technological and Water Use Scenarios

In order to assess the influence of the technology used and the rate of development adopted, we defined two scenarios which are representative for the highest and lowest values (in terms of potential environmental impact) of a range of variables characterizing the development of a shale play. These ‘high’ and ‘low’ scenarios also include several parameters which affect the efficiency and total amount of water used. All variables used are summarized in Table 1. The assumed lifespan of the well pads is 10 years in both cases.

**Table 1:** Specific technology and water use variables employed for the high and low development rate scenarios

Technology	High	Low
Well pad size (size during construction)	1.06 ha (3.55)	3.75 ha (9.93)
Number of wells per pad	2	16
Well pad spacing	8 2-well pads/256 ha	16-well pad/1036 ha
Nr. well pads placed per 5 years	294	37
Flowback (%)	0	70
Recycling scenario (%)	0	70
Consumption ratio (%)	100	51
Water consumption per well (m^3^)	19,000	8000

We consider both the total amount of freshwater withdrawn for use in the shale gas extraction process (the majority of which is used for fracking), and the share thereof which is ‘consumed,’ i.e., either evaporated, infiltrated into the ground or polluted to an extent that it cannot be directly re-used during the fracking process. The actual amount of water used for shale gas extraction by hydraulic fracturing varies greatly (Sumi 2008; DGIP 2011; Clark et al. 2013), depending on several factors:

- Local geology (depth, dimensions of shale play, permeability, and type of shale)
- Technology used (can allow more efficient use of water and reduce leakages)
- Flowback (amount of water recovered after fracking)
- Recycling ratio (how much of the water used in fracking is directly re-used on-site)
- Number of fracks carried out per well
- Duration of drilling

**Fig. 3:**
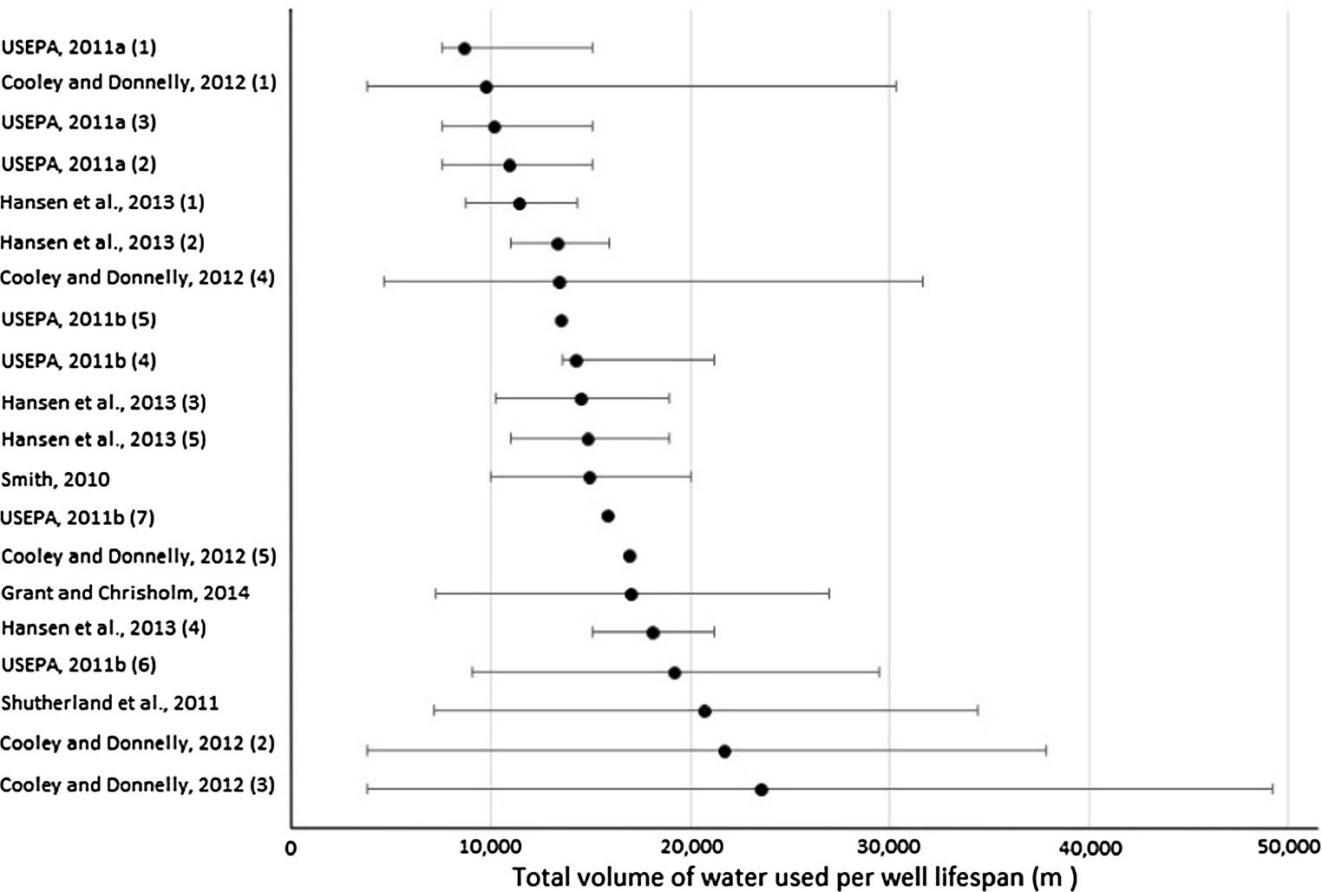
The range of water use estimates for shale gas extraction, with minimum, maximum, and average values shown for the various studies considered in m^3^ per well lifespan

We reviewed the available literature from 2011 onwards to assess the range of estimated volumes of water required for a single well. Only the most recent estimates were taken into account to reflect the current technology and water use efficiency. The values used to estimate the average water requirements are shown in Fig. 3  (based on Cooley and Donnelly 2012; Grant and Chisholm 2014; USEPA 2011a, b; Hansen et al. 2013; Smith 2010; Sutherland et al. 2011). Estimated water demand ranges from as low as 3500 m^3^ to almost 50,000 m^3^ per well (over the whole lifespan), with an average range in water requirements between 8000 and 19,000 m^3^. We use these values to represent the required volumes for our low and high impact scenarios, respectively.

Upon completion of the fracking process, the direction of fluid flow reverses, with a proportion of the injected fluid returning to the surface. This “flowback” usually ranges from 5 to 50 % of injected freshwater (Sumi 2008; NYSDEC 2011; DGIP 2011), and in some cases may even reach up to 70 % (King 2012). Flowback water may also potentially be recycled, hence reducing cumulative freshwater demands. Gaudlip and Paugh (2008) suggest a recycling rate for flowback water of 70 % in Pennsylvania for best-performing companies, and up to 71.5 % was measured for the Marcellus shale in 2011 (Maloney and Yoxtheimer 2012). This said, on average in the US only some 6–10 % of total water used in fracking is recovered and re-used on-site (Mantell 2011). For the high impact scenario, we therefore assume there to be no recovered or recycled flowback water. In the low impact scenario, we assume a maximum flowback of 70 %, of which 70 % is recycled on-site, so reducing the total amount of water consumed by 49 %.

In the case of Poland, the use of groundwater resources up to 1–2 km deep is permitted (Uliasz-Misiak et al. 2014). Since we lack data on the potential source of water for use in fracking, we assume the same shares as for industrial purposes per catchment. This means that on average for our study area we assume 28 % of the water for fracking to be withdrawn from groundwater resources, and the remaining 72 % from surface water bodies. Since the assumed lifespan of the well pads is 10 years, we divide their water use over two of the 5-year time steps. We assume the water use to be proportional to the gas production, so divide the share of water use according to the production curve presented in Broderick et al. (2011). Seventy percent of the total water use per well pad is therefore allocated in the first time step, and thirty percent in the following time step. This amount was then divided by 5 to estimate the actual amount of water required for 1 year to ensure comparability with the competing water uses (which are calculated annually).

#### Legislative Scenarios

In addition, a further two scenarios were developed, one based on the current legislation in place and the other representing a potential future legislation which is much more restrictive. The purpose of using these two scenarios in addition was to assess the possible influence that adopting different legislative frameworks may have. In the case of the Marcellus and Utica shales in the US, the amount of water withdrawn for shale gas extraction is regulated. Any surface or groundwater withdrawals exceeding 1,00,000 gallons (378.5 m^3^) per day require approval from the specific river basin commission (Arthur et al. 2009). Freshwater resources are protected in Poland, although the extent to which varies on a case-by-case basis. There may, for example, be restrictions on the amount of water which can be extracted from a source. Our current legislative scenario excludes shale gas exploitation directly adjacent to water bodies, and in areas potentially at risk of a 100-year return period flood. In addition to this, the restrictive scenario excludes a buffer area of 200 m around all water bodies and waterways. An overview of the assumptions made for the scenarios is given in Table 2. These restrictions are applied at each modeling time step to exclude areas where no well pads can be placed.

**Table 2:** Summary of excluded areas according to the current and restrictive legislative scenarios defined

Constraint	Current	Restrictive
Nature reserves	Total area	Total area, 200 m setback
Other natural protected areas	–	Total area
Flooded areas	Total area	Total area
Inhabited areas	Urban and industrial areas	Any occupied buildings, 200 m setback
Road network	50 m each side	50 m each side
Railways	50 m each side	50 m each side
High voltage lines	50 m each side	50 m each side
Caves and caverns	–	Total area, 300 m setback
Mines	–	Total area, 300 m setback
Historic gas/oil wells	–	Total area, 300 m setback
Water wells	–	Total area, 500 m setback
Aquatic habitats	–	Total area, 200 m setback

### Water Quantity Assessment

The water use modeled for each shale gas development scenario was compared to a baseline scenario which excluded any potential shale gas extraction activities. The water use model used (Vandecasteele et al. 2013, 2014) estimates water withdrawals and consumption for the public, industrial, and agricultural sectors. It computes water withdrawals using the reference year 2006, and can forecast to 2030 using various data projections. The methodology is based on the disaggregation of water use statistics to the appropriate land use classes using proxy data. The main statistical data source for Poland was the “Environment 2011” report from the Central Statistical Office of Poland (CSO 2011), which gives water withdrawals for the public, industrial, and agricultural sectors at river basin level. For all sectors, water consumption maps were calculated as a fraction of the withdrawal maps (Vandecasteele et al. 2013). We assumed 20 % of water used for the public supply to be consumed; 15 % of industrial water, and 75 % of agricultural water (mostly used for irrigation). The source of freshwater was also indicated per catchment. On average for our study area, 91 % of public supply is withdrawn from groundwater resources, whereas 72 % of industrial water is withdrawn from surface waterbodies. Due to a lack of data, we assumed agricultural water to be withdrawn from surface resources.

We assume the water used for fracking to be extracted within the same river catchment where the drilling takes place, taking into account that natural gas companies will try to minimize transport costs, which in some cases may exceed the actual cost of the water itself (Arthur et al. 2009). The impact of additional water use for shale gas extraction for the different scenarios is therefore assessed at the river catchment scale, using the water exploitation index (WEI). The index is the ratio of total water withdrawals to the total amount of water available, and can be calculated for both the total amount of water abstracted (WEIabs), and the total amount consumed (WEIcns). We used our water withdrawal and consumption maps in conjunction with the average annual surface and ground freshwater availability to compute both indicators. The WEIcns was also used as a suitability factor to determine where shale gas extraction should be situated in the modeling process (Baranzelli et al. 2014). Where the water exploitation was already high, suitability was decreased, hence discouraging shale gas extraction in that river basin. We compute all water withdrawal and consumption maps and the WEIabs and WEIcns every 5 years, starting from the initial year of possible extraction—2015. The initial baseline indicators for 2015 serve to help define the optimal location for the first well pads. In the subsequent time steps, the indicators are re-calculated for each scenario, allowing us to analyze the spatial and temporal effect of the additional water abstractions required for the shale gas extraction on the state of the available water resources.

### Water Quality Assessment

Several issues need to be addressed to ensure that shale gas can be produced in a manner that meets environmental and public health protection goals (Howarth and Ingraffea 2011). Since hydraulic fracturing typically involves the use of large quantities of water and chemicals, associated risks for contamination of ground and surface waters, along with environmental and human health impacts, require careful consideration. In the present paper, we focus on water-related impact. Nevertheless, concern for both ecosystems and human health (both occupational and for the general population) due to chemicals used in shale gas development should be evaluated. Ideally, the assessment should entail the evaluation of:

- Emissions (quantities and ratios of water, proppants, and chemicals; operational/accidental releases; injected chemicals/formation chemicals)
- Exposure (fate of the chemicals when emitted into air, water, and soil; exposure pathways for ecosystems and humans)
- Effects (toxicological endpoint of both the injected and the formation chemicals)

**Fig. 4:**
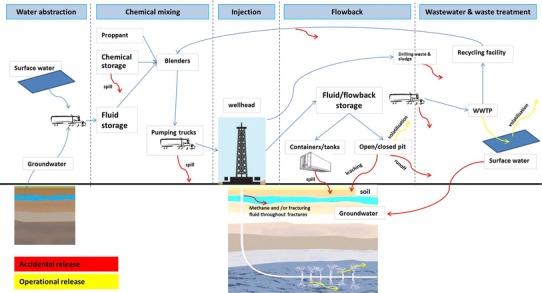
Conceptual model of accidental and operational releases of chemicals in shale gas fracking. *WWTP* waste water treatment plants

The release of fracking chemicals into the environment may occur under two circumstances: as operational releases (due to the specific processes associated with shale gas development) or as accidental releases. Moreover, two typologies of chemicals should be considered: the chemicals that are injected into the well (injected chemicals) and formation chemicals that are mobilized from the fractured formation and brought to the surface in flowback water. The latter may include heavy metals (some of them particularly toxic (e.g., Hg or Cr)), salts, and radionuclides (Kargbo et al. 2010). The key steps in hydraulic fracturing where operational and/or accidental release of chemicals may occur are indicated in Fig. 4.

Both operational and accidental emissions to air, soil, and both surface and groundwater may occur at several stages in the extraction process, including during storage and transport of chemicals and fracking fluid. This is due to the volatilization of specific chemicals, spillages, and infiltration from surface ponds to soil and groundwater stores. Waste water is either treated on-site, re-injected into the rock mass (Rahm 2011), or transported to (usually industrial) treatment plants. In Poland, discharge to sewage requires a permit, but discharge to industrial waste treatment plants is allowed to some extent (Uliasz-Misiak et al. 2014).

We undertook a screening-level assessment of the potential impacts on water associated with a subset of chemicals recorded in the literature as being currently used in the hydraulic fracturing of shale gas wells. Even though the Polish Environmental Protection Law states that the composition of fracking fluid is not confidential (Uliasz-Misiak et al. 2014), detailed reports of specific chemicals used in Poland are scarce. We therefore based our analysis on a list of over 1000 chemicals used in fracking, as reported by USEPA (2012a) (this list is given in the supplementary information). In order to assess the potential fate of these chemicals in the environment, we needed to (i) identify the processes involved which may incur emissions; (ii) gather data on the physicochemical properties of the chemicals; and (iii) run multimedia fate model. The physicochemical properties were calculated using the EPIsuite^TM^ (Estimation Programs Interface) model version 4.11^1^ (USEPA 2012b). This is a Windows^®^-based suite of physicochemical property and environmental fate estimation programs, developed as a screening-level tool, from which we took the physicochemical properties only. Among other results, the model provides two partition coefficients (Kow—partition octanol–water and Kaw—partition water–air), which were used to define the chemical space of the chemicals potentially involved in fracking.

Additionally, the environmental fate and potential harm to freshwater ecosystems and human health were assessed using the multimedia model USEtox (Rosenbaum et al. 2008). USEtox was used to conduct a screening-level assessment of the potential impact of the substances based on different routes and pathways of release. USEtox incorporates a matrix framework for multimedia modeling, allowing the separation of fate, exposure, and ecotoxicity effects in the determination of an overall Characterization Factor (CF). In fact, Usetox includes three basic components: fate factors (FF); exposure factors (XF); and effect factors (EF), which are combined (multiplied) to give a result in comparative toxic units (CTUs). The resulting CTU will therefore be higher with any increase in residence time, higher exposure factor or higher effect factor. An overview of the different components of the USEtox model is given in Table 3.

**Table 3:** Description of input data and factors for the screening-level assessment of potential impact on freshwater, based on the USEtox manual (Huijbregts et al. 2010)

Model component	Description
Physicochemical properties	USEtox physicochemical data, available for calculating FF, XF, and EF
Fate factors (FF)	Multimedia box model USEtox, fate component. The fate factor is equal to the compartment-specific residence time (in days) of a chemical in the environment. The residence time of a chemical depends on (i) the properties of the chemical, (ii) the selected emission compartment (e.g., urban air), and (iii) the selected receiving compartment (e.g., fresh water at the continental scale). The fate component of USEtox accounts for the removal and intermediate transport processes of chemicals in the environment
Exposure factors (XF)	The environmental exposure factor for freshwater ecotoxicity is the fraction of a chemical dissolved in freshwater (FRw.w). This takes into account: the partition coefficient between water and suspended solids (l/kg); suspended matter concentration in freshwater; partitioning coefficient between dissolved organic carbon and water; dissolved organic carbon concentration in freshwater; the bioconcentration factor in fish, and the concentration of biota in water
Effect factors (EF)	The ecotoxicological effect factor is calculated by determining the linear slope along the concentration–response relationship up to the point where the fraction of effected species is 0.5. Aquatic ecotoxicological effect factors are based on geometric means of single species EC50 test data. Chronic values have priority as long as they represent measured EC50 values. Second-order priority is given to acute data, applying an acute-to-chronic extrapolation factor that is set to a default factor of 2. All available data have been used, meaning that for each substance, the derived SSD is based on a different number of species. The data are calculated using the AMI method (Payet 2005), where the final geometric mean is obtained by calculating the geometric of (1) all data for the same species; (2) all species belonging to the same phyla, and (3) between different phyla. This has the advantage of giving a final result less influenced by extremes values. As a consequence, the calculated HC50 is an average of all data
Comparative toxic units (CTU)	The characterization factor for aquatic ecotoxicity impacts (ecotoxicity potential) is expressed in comparative toxic units (CTUs), and represents an estimate of the potentially affected fraction of species (PAF) integrated over time and volume, per unit mass of a chemical emitted. Hence, the unit is mass-based and is [CTU per kg emitted] = [PAF × m^3^ × day per kg emitted]

The resulting CFs (expressed as CTUs) were calculated accounting for potential emissions into water, soil and/or air of a unit of chemical (e.g., 1 kg). As we miss specific information of quantities emitted, our calculation leads to a prioritization of chemicals assuming an equal unit of emission for all of them. Assuming a linear dose–response function for each disease endpoint and intake route, the ecotoxicity effect factor was calculated as 0.5/ED50, where ED50 is the lifetime daily dose resulting in a probability of effect of 0.5.

## Results

### Competing Water Uses and Exploitation of Freshwater Resources

**Fig. 5:**
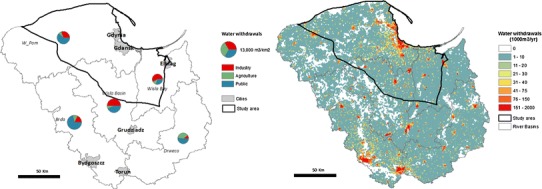
Water withdrawals for 2012, given by **a** sector and major catchment area, and **b** at 1 km resolution using the water use model

The water withdrawn for sectoral use is given per catchment and per sector (Fig. 5a, based on statistics obtained from CSO 2011). The industrial sector accounts for the greatest share of water withdrawn in the Wisla Bay and Wisla Basin (up to the Brda catchment). In the remaining catchments, the greatest share is withdrawn for use in the public water supply. The total amount of water withdrawn per km^2^ remains relatively constant, with the largest amounts being withdrawn in the Brda and Wisla basins. Figure 5b shows the total water withdrawals for 2012, calculated using the same statistics and applying our water use model at 1 km resolution.

According to the simulations carried out with the water use model, there is a steady increase in total water withdrawals from 2015 (119.8 hm^3^) to 2030 (148.5 hm^3^) due to a growing population and increasing industrial production. Table 4 gives the total amount of water used in shale gas extraction as a share of the total water withdrawals for each step of the high and low impact technological scenarios. The total water use in the study area is not directly influenced by the type of legislation put in place, so only the differences between the technology scenarios are shown.

**Table 4:** Total amount of water withdrawn for use in shale gas extraction compared to the total water use per scenario (both in hm^3^ and in  % of the total water used within the study area)

Year	Total water withdrawals (hm^3^)	Low (hm^3^)	High (hm^3^)	Low (% of total)	High (% of total)
2015	119.796	0.041	0.782	0.03	0.65
2020	130.717	0.059	1.123	0.05	0.86
2025	138.927	0.059	1.123	0.04	0.81
2030	148.471	0.059	1.123	0.04	0.76

If we consider only the water withdrawn within the shale play area, the share of water use for shale gas extraction accounts for up to 0.05 and 0.86 % of the total water withdrawals for all sectors for the low and high impact scenarios, respectively. This is consistent with values found in the US (Table 5, (MIT 2011)).

**Table 5:** Comparative water usage in major shale plays (MIT 2011)

Shale gas plays	Public supply	Industrial/mining	Irrigation	Livestock	Shale gas
Barnett, TX	82.7 %	3.7 %	6.3 %	2.3 %	0.4 %
Fayetteville, AR	2.3 %	33.3 %	62.9 %	0.3 %	0.1 %
Haynesville, LA/TX	45.9 %	13.5 %	8.5 %	4.0 %	0.8 %
Marcellus, NY/PA/WV	12.0 %	71.7 %	0.1 %	<0.1 %	<0.1 %

**Fig. 6:**
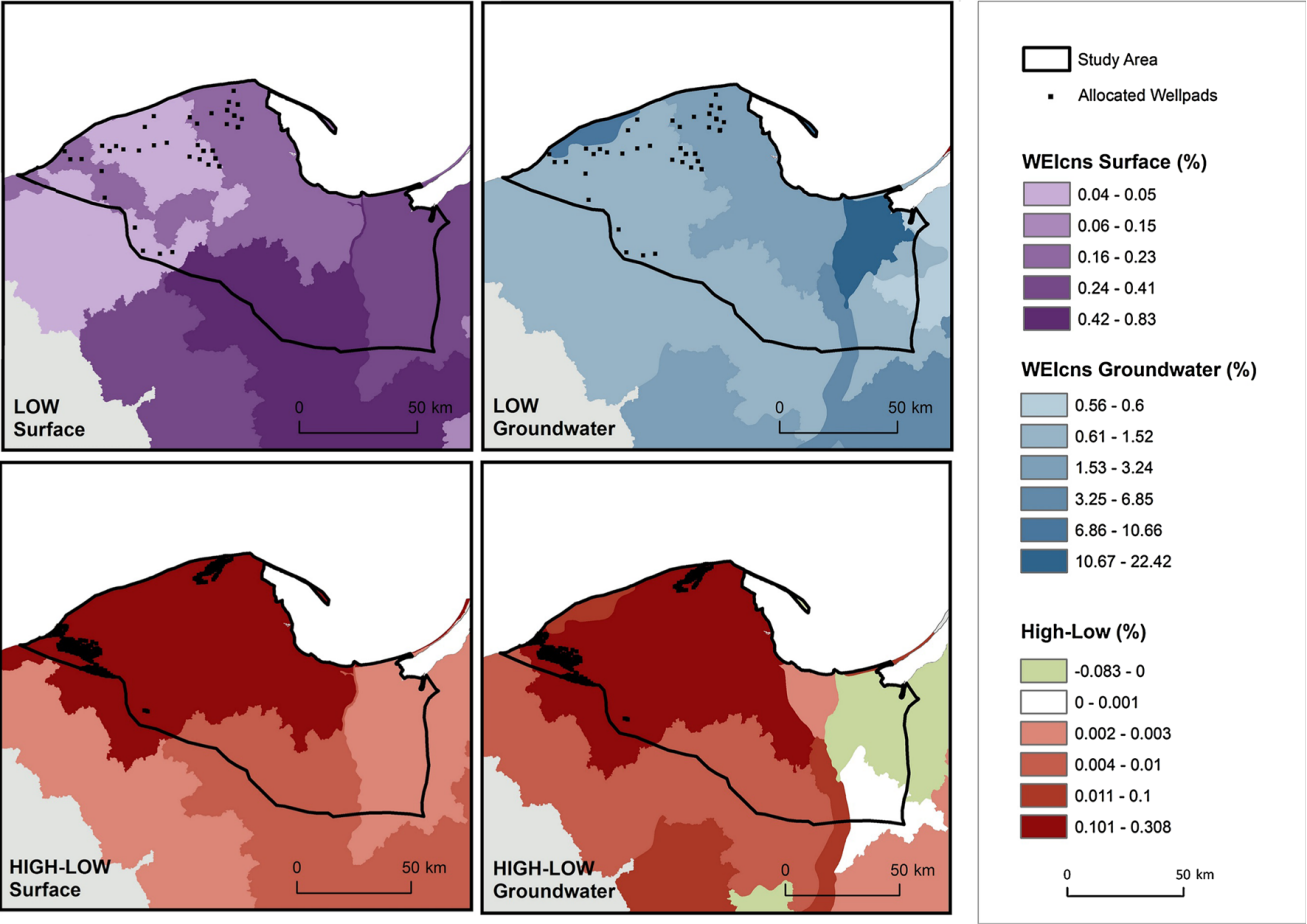
The Water Exploitation Index for consumption (WEIcns) for surface and groundwater for 2025 for the lowest impact compared to the highest impact scenario

The WEIcns for surface and groundwater resources was calculated for each scenario. Figure 6 shows the WEIcns for the lowest and highest combined impact scenarios, respectively (LOW = lowest impact technological scenario with restrictive legislative framework; HIGH = highest impact technological scenario with current legislative framework). The figure is overlain with the well pads allocated in 2025 for each scenario combination for comparison.

The increase in impact seen within the shale play corresponds directly to the placement of the well pads. There is also a greater impact on the overall WEIcns calculated for the high combined scenario. Although this difference accounts for only up to a 0.3 % higher WEIcns at the catchment scale, due to the much higher density of well pad placement in the high scenario (shown in the bottom panels for comparison), there will be a much higher impact locally. Changes seen outside the shale play area between scenarios can be attributed to the differing land use maps simulated per shale gas extraction scenario. Depending on where well pads are placed, urban and industrial land may be correspondingly increased or decreased in other regions to compensate and meet the demands which are built into the land use model. This differing land use results in altered water use maps, which in turn directly impact the calculation of the WEI. In the high scenario, the WEIcns for surface water reaches a maximum of 0.83 %, and the WEIcns for groundwater 22.42 %. This indicates that due to the higher impact of withdrawals from groundwater on the overall exploitation, water for shale gas extraction should preferentially be withdrawn from surface water bodies.

### Screening Assessment of Potential Impact of Chemicals on Freshwater

**Fig. 7:**
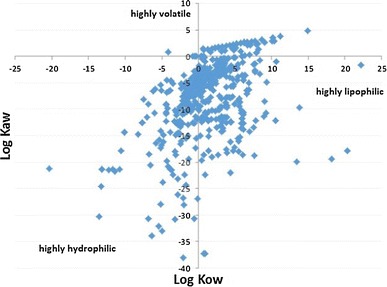
Position of the chemicals used in fracking (as listed by USEPA 2012a) in the chemical space defined by Log Kow and Log Kaw

The EPIsuite^TM^ model was run to calculate the physicochemical properties of the list of over 1000 chemicals provided by USEPA (2012a). The distribution of these chemicals in the chemical space defined by the partitioning coefficients Kow and Kaw is reported in Fig. 7. The considerable heterogeneity in physicochemical properties shown—ranging from highly volatile to strongly lipophilic and hydrophilic—highlights that they may follow very different pathways in the environmental fate. It should be noted that, beyond a screening assessment, additional information on chemical properties is needed to further assess the potential fate of chemicals. Log Kow may have limited value for the estimation of environmental fate of chemicals ionized across environmentally relevant pH, which influences bioavailability, partitioning to soils, sediments, organisms, and so on. It has been estimated that one-third of the chemicals registered under REACH are ionizable (Franco et al. 2010). This is a problem affecting several multimedia models including those used in the present study (EPISuite and USEtox), which are optimized for neutral hydrophobic substances (Rosenbaum et al. 2008). Efforts to develop models suitable for ionizing substances are on-going (Van Zelm et al. 2013; Franco and Trapp 2010). However, multimedia model adaptations for accounting for ionizable chemicals typically result in higher freshwater fate factors for ionized acids (p*K*a < 7), while for ionized bases (p*K*a > 7), larger as well as smaller fate factors are seen. For acids and bases that are less than 50 % ionized in freshwater, the changes in fate factors are relatively small (<10 %) (Van Zelm et al. 2013). Additionally, site-specific aspects may greatly influence the fate in real water bodies (see e.g., Valenti et al. 2011). Nonetheless, accounting for the above-mentioned limitation, the chemical space covered by the substances demonstrates the need for the proper modeling of chemicals which are very diverse in terms of physicochemical properties and potential fate in the environment.

Applying USEtox, the results were expressed in comparative toxic units (CTUe), which provide an estimate of the potentially affected fraction (PAF) of species integrated over time and volume per unit mass of a chemical emitted (PAF m^3^ day kg^−1^). The results highlight wide variability in terms of potential impacts for ecosystems and human health. For example, when chemicals are emitted directly to water, there is tremendous variability in potential impacts (over 12 orders of magnitude). This could be due to the fact that the fate, toxicological properties, and potential harmfulness of the substances are very diverse. When emitted in water, the chemicals that tend to remain in water imply higher CTUe, whereas those that volatilize or are adsorbed by the sediments result in lower CTUe values. These results should be taken with caution, as the fate, the exposure and the effect components may be affected by the presence of ionisable compounds (e.g., for emission into freshwater, the ratio between FF accounting for ionization or assuming neutral substance varies from 0.24 to 1.6; for emission into air, from 0.058 to 6000; Van zelm et al. 2013).

**Fig. 8:**
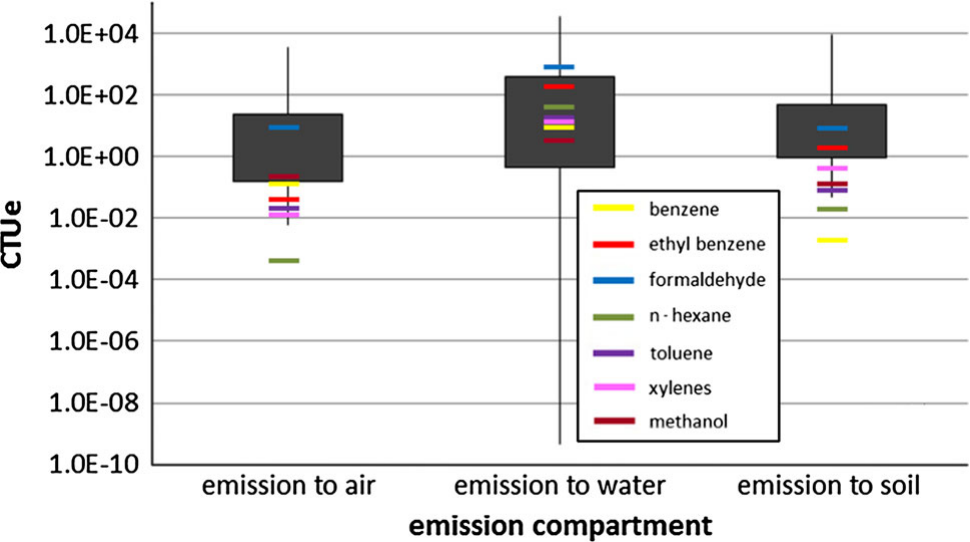
Comparative toxic units (CTUe) for aquatic ecotoxicity, assuming an emission into air, water, and soil of 250 out of 930 chemicals listed in USEPA (2012a, b). The graph refers to four percentiles, namely 5, 25, 75, and 95th

Figure 8 reports the comparative toxic unit for ecotoxicity for all the substances reported by USEPA (2012a), highlighting those frequently mentioned in the literature as main emissions coming from shale gas (e.g., benzene, toluene, ethylbenzene, and xylenes (BTEX), for which values are given in Table 6). Notably, irrespective of the route of the emission, the potential ecotoxicological concern for freshwater related to many chemicals is high, even beyond the value reported for substances already known as being of concern for emission to air and water directly. In the case of emission to soil, the chemicals of concern are relatively few (due to high volatilization, the relative contribution to freshwater ecotoxicity through soil emission is low).

**Table 6:** CTUe for selected chemicals considered already in the literature as chemicals of concern for the environmental impact of shale gas extraction

Chemical	Emission to air	Emission to water	Emission to soil
Benzene	6.40E − 02	6.60E − 01	1.23E − 03
Ethyl benzene	2.84E − 02	1.75E + 02	1.70E + 00
Formaldehyde	2.68E + 01	2.97E + 02	8.40E + 01
*n*-hexane	1.35E − 04	6.48E + 01	2.44E − 02
Toluene	1.28E − 02	5.59E + 01	6.96E − 01
Xylene	1.06E − 02	7.74E + 01	8.56E − 01
Methanol	2.24E − 01	2.65E + 00	5.11E − 01

Effect factors are calculated using the AMI method (Payet 2005, as explained in Table 3). This method ensures that the final result is less affected by extreme values.

## Discussion

It should be noted that both for the quantity and quality assessments, there were several limitations due to data availability and some assumptions had to be made. For example, although this was not taken into account, wells may also be repeatedly fracked to maximize productivity (Berman 2009; NYSDEC 2011; Ineson 2008). Irrigation water is assumed to be withdrawn from surface water, but may actually in part be extracted from groundwater resources in some cases—this would mean that the stress on groundwater resources is underestimated. The actual situation in terms of water use may also be very different in Europe compared to that in the United States. Indeed, the often-repeated assertion that European shale plays tend to be deeper and more complex suggests that water demands may also be different. This study would therefore benefit from additional European data for the definition of the scenarios as it becomes available.

It should also be stressed that the results of the screening chemical assessment are meant as a screening since the exact products and quantities were missing. Impacts may vary greatly according to spatial and temporal aspects and site-specific contexts. Although this was not possible here due to data limitations, the evaluation should therefore ideally be site-specific (e.g., Vidic et al. 2013). Due to the wide variety of chemicals used and the heterogeneity of their physicochemical properties and associated toxicological concerns, a detailed human and ecological risk assessment is recommended, covering different endpoints and possible targets of impact. Additionally, improving knowledge related to the chemicals potentially involved in both operational and accidental releases is essential. It is also important to note that this analysis has focused on injected chemicals only, whereas formation chemicals may also pose environmental and human health concerns.

The screening assessment undertaken here, along with a review of existing studies, supports the following recommendations (complementary to those provided by Colborn et al. (2011)) for an optimal management and prevention of impacts:

- *Reporting* (i) each drilling and fracking operation, as well as total fluid injected; (ii) chemicals used and quantities employed over time; (iii) the level of treatment of flowback and produced water; (iv) potential mixtures of chemicals that may occur in the event of operational or accidental releases; (v) local context, including the geology/hydrogeology and climatic aspects (Ciuffo and Sala 2013);
- *Assessing* (i) impacts at different scales: local, regional, and global; (ii) the comprehensive life cycle of shale gas exploitation, from shale play preparation to closure and mid-term/long-term impacts in order to avoid burden shifting between operational stages or impact categories;
- *Policy Synergies* Elucidation of the role of the REACH Directive implementation (EC 2006) in the systematic accounting of chemicals used in shale gas exploitation and their related physicochemical and toxicological properties, as already started by Gottardo et al. (2013).

Water handling is estimated to account for some 10 % of the operational cost of a well (Gay et al. 2012), making it an important issue to be addressed by operators. This includes not only the need for management of issues related to water availability but also optimization of disposal, treatment, and transport.

The efficiency of water use in fracking has been noted to be increasing over the last 10 years (Nicot et al. 2014). There have also been several initiatives to substitute fresh water with brackish or even saline water. In Texas, for example, increased pressure on water resources and regional droughts have encouraged the use of brackish groundwater (Standen 2012; Ghahremani and Clapp 2014), which now accounts for up to 30 % of the water used for fracking. The pre-treatment and extraction thereof (in the case of groundwater) are, however, still a major expense. The use of alternative substances to water is also being developed (Rogala et al. 2013; Gandossi 2013), for example, a gelled form of liquid petroleum gas (LPG) used which may even have a higher recovery rate of shale gas (Wilson 2013).

Even though new technologies are being developed for on-site treatment and recycling of flowback water (Miller et al. 2013), produced water is still mostly re-injected into the ground via wells (Nicot et al. 2014). Some of this flowback water may potentially be recovered after desalinization for alternative uses (Shaffer et al. 2013). However, the proper disposal of non-recycled produced water remains a concern.

## Conclusions

The methodology used aimed at assessing the range of possible impacts of shale gas extraction on water resources within the current data limitations. Therefore, even though some important conclusions can be drawn, care should be taken in the interpretation of the results.

The scenarios modeled vary greatly in terms of projected water withdrawals and consumption. We took an estimated range of water use per well between 8000 and 19,000 m^3^, and assumed that in the best case scenario, 70 % of flowback water would be recovered, of which up to 70 % could be recycled, meaning an overall re-use of 49 % of water—in the worst case scenario, we assumed all water to be lost directly to the environment. In the best case, water use for shale gas accounted for only 0.03 % of the total water use for all sectors within the shale play. In the worst case scenario, this proportion rose to 0.86 %, at which point there may be impacts seen locally, especially where there are already water shortages or periods of drought. The wide range in estimated absolute water use per scenario stresses the importance of water use efficiency to reduce the overall impact on the direct environment. The share of water use attributed to shale gas extraction locally was comparable to those found in the major shale plays in the USA (Table 5). The WEIcns was also seen to vary considerably according to the scenario and the maximum in the last calculated time step (2028) was 0.83 % for surface water, and 22.4 % for groundwater. This highlights the importance of the source from which water is extracted—for the scenarios run, we assumed on average 28 % of fracking water to originate from groundwater resources, but in fact, this amount should be minimized due to the limited availability of groundwater compared to surface resources.

The study showed that additional pressure would be put on local water resources due to future shale gas extraction. The extent to which the development of this resource will impact on the direct aquatic environment varies greatly with the rate of extraction, the technology used, and especially the efficiency of water use and the recycling thereof. Maximizing the recovery of water as flowback, and increasing the recycling ratio would reduce the absolute water requirements per well, and reduce both the impact on the environment and the cost of transport for the companies involved.

In the screening-level risk assessment carried out, physicochemical properties were estimated with EPISuite^TM^ (suited for screening analysis only), the fate in the environment was assessed using a box model (USEtox), and the effects were evaluated only for freshwater and adopting EC50 as endpoint. Beyond current limitations, the analysis performed allows the identification of some important elements. The evaluation highlighted that many of the chemicals used may pose ecosystem health risks. Of special concern is the heterogeneity of the chemicals and their physicochemical properties, meaning they may propagate and persist in all mediums (not just water but also air and soil). This in turn leads to a wide range of associated toxicological concerns. Some of the chemicals present in the list of those used in fracking (e.g., BTEX) are of very high concern not only for drinking water (Gross et al. 2013; Swanson and Krause 2011), but also for ecotoxicity-related impacts. This is of particular importance if we consider that some of those chemicals present a potential risk higher than those of the substances usually monitored or reported in the literature for shale gas.

Figure 7 indicates the numerous stages in the shale gas extraction process where there may be accidental or operational release of substances (both wastewater and chemicals directly), and where special attention should be paid to the reduction of accidental losses, and the proper regulation of operational releases. Concerning the possible contamination of water due to shale gas exploitation, the results clearly showed that there is a need to further integrate risk assessment and life cycle assessment methodologies in the analysis of the environmental risk associated to shale gas development, as in Mangmeechai et al. 2013.

The more efficiently water can be used, and the higher the flowback and recycling ratio achieved, the lower will be the overall impact on freshwater resources, also in terms of water quality considerations.

## Electronic supplementary material

Below is the link to the electronic supplementary material.

Supplementary material 1 (XLS 1374 kb)

## Footnotes

1 The EPI (Estimation Programs Interface) Suite™ is a Windows^®^-based suite of physicochemical property and environmental fate estimation programs. EPI Suite™ is a screening-level tool and should not be used if acceptable measured values are available.
